# Unveiling viral diversity and dynamics in mosquitoes through metagenomic analysis in Guizhou Province, China

**DOI:** 10.1186/s40249-025-01321-9

**Published:** 2025-06-19

**Authors:** Yan Linghu, Rui-Si Hu, Xiao-Min Tang, Rong-Ting Li, Wei-Yi Li, Jia-Hong Wu

**Affiliations:** 1 Characteristic Key Laboratory of Modern Pathogen Biology, School of Basic Medicine, Guizhou Medical University, Guiyang, 561113 Guizhou Province People’s Republic of China; 2 Key Laboratory of Intelligent Medicine and Health Data Science, College of Medicine, Sichuan University of Arts and Science, Dazhou, 635000 Sichuan Province People’s Republic of China; 3 Guizhou Provincial Center for Disease Control and Prevention, Guiyang, Guizhou Province People’s Republic of China; 4 Weining Yi, Hui, and Mao Autonomous County Center for Disease Control and Prevention, Bijie, Guizhou Province People’s Republic of China; 5 Department of Human Parasitology, School of Basic Medicine, Guizhou Medical University, Guiyang, Guizhou Province People’s Republic of China; 6 Guizhou Key Laboratory of Microbio and Infectious Disease Prevention and Control, Guiyang, Guizhou Province People’s Republic of China

**Keywords:** Mosquito, Viral metagenomics, *Aedes albopictus*, Japanese encephalitis virus, Getah virus, Banna virus, Guizhou, China

## Abstract

**Background:**

Poverty, disease, and vector ecology intersect to present ongoing health threats, particularly in ecologically sensitive regions. Guizhou Province in China, with its complex karst topography and rich biodiversity, offers a unique environment to study mosquito-borne viral transmission. Despite over 5000 reported cases of Japanese encephalitis in the past two decades and the detection of Zika virus in 2016, the virological landscape of this region remains poorly understood. This study aims to characterize the mosquito-associated virome, assess viral diversity, and identify factors influencing transmission dynamics in Guizhou Province.

**Methods:**

Between 2021 and 2022, we conducted a 2-year mosquito surveillance across eight ecologically distinct regions in Guizhou Province. Adult mosquitoes were collected using a variety of methods, including BG Mosquitaire CO_2_ traps, mosquito-killing lamps, manual collection, human bait traps, and oviposition traps. To investigate the virome diversity and dynamics within mosquito populations, we performed metagenomic sequencing and bioinformatics analysis on pooled mosquito samples collected from geographically diverse sampling sites.

**Results:**

We collected more than 40,000 adult mosquitoes, primarily belonging to four genera: *Aedes*, *Anopheles*, *Armigeres*, and *Culex*. Dominant species included *Aedes albopictus*, *Anopheles sinensis*, *Armigeres subalbatus*, and *Culex tritaeniorhynchus*. Notably, we report the first provincial record of the *Anopheles baileyi* complex, expanding the known distribution of mosquito vector in this region. Viral metagenomic sequencing, coupled with bioinformatic analysis, identified 162 viral contigs, including 140 known and 22 previously uncharacterized viruses. We experimentally confirmed the genotypes of three medically important zoonotic viruses: Japanese encephalitis virus (JEV-GI), Getah virus (GETV-GIII) and Banna virus (BAV-A2). Comparative analysis of viral abundance across mosquito species revealed that *Aedes albopictus* populations in Guizhou harbor a distinct virome composition, diverging from those reported in other geographic regions.

**Conclusions:**

This study presents the comprehensive characterization of the mosquito-associated virome in Guizhou Province, providing critical insights into viral diversity, vector competence, and transmission dynamics within karst ecosystems. The detection of multiple zoonotic viruses highlights the need for strengthened surveillance and targeted public health interventions in this region.

**Supplementary Information:**

The online version contains supplementary material available at 10.1186/s40249-025-01321-9.

## Background

Mosquitoes are the most significant arthropod vectors worldwide, substantially increasing the burden of infectious diseases and putting nearly half of the world’s population at risk, particularly from mosquito-borne viral infections [1, 2]. The mosquito virome comprises insect-specific viruses (ISVs) and mosquito-borne viruses (MBVs). ISVs are restricted to arthropods, while MBVs can replicate in mosquitoes and pose zoonotic risks to humans and mammals [3, 4]. Key vectors include *Aedes aegypti* and *Ae. albopictus*, which transmit dengue virus (DENV), chikungunya virus (CHIKV), and Zika virus (ZIKV), associated with symptoms such as high fever, rash, hemorrhage, and joint pain [5–7]. In contrast, *Culex quinquefasciatus* and *Cx. tritaeniorhynchus* transmit West Nile virus (WNV) and Japanese encephalitis virus (JEV), which are linked to clinical manifestations such as fever, headache, and encephalitis [8, 9]. Amid global climate and environmental changes, the growing threats of mosquito-borne diseases underscores the urgent need for comprehensive virome surveillance [10, 11]. Such efforts are essential for identifying MBVs, tracking their evolution, and detecting emerging zoonotic threats that could pose significant public health challenges.

Guizhou Province, a karst-dominated region covering 73.8% of its area in southwestern China, not only supports rich biodiversity but also provides ideal conditions for mosquito breeding and MBV transmission. These ecological vulnerabilities are exacerbated by long-standing regional development disparities, leading to Guizhou being labeled a “spatial poverty trap” [12, 13]. In recent years, the Poverty Alleviation Relocation initiative has driven the cross-regional migration of approximately one-fifth of the population, intensifying human mobility and posing new ecological management challenges [14]. Coupled with the karst environmental features such as water accumulation points, high humidity, and biodiversity, these dynamics may facilitate mosquito proliferation and increased the risk of cross-species MBV transmission. Over the past two decades, Guizhou has reported 5138 cases of Japanese encephalitis and 152 related deaths [15], as well as the detection of ZIKV in local mosquitoes in 2016 [16], underscoring the growing burden of mosquito-borne diseases. Despite these risks, systematic and up-to-date studies on the ecological and epidemiological dynamics of MBVs remain critically lacking.

Viral metagenomics is a high-throughput approach that enables comprehensive identification of transcriptional characteristics and regulatory patterns of viral genomes within a given pool of mosquito samples [17]. This methodology has been increasingly adopted in recent studies to investigate the viral metagenomics of various mosquito genera across diverse geographic regions, uncovering significant diversity among both known and novel viruses [18–21]. Beyond characterizing viral diversity, viral metagenomics serves as a powerful tool for elucidating the genomic evolution via identifying adaptive mutations, recombination events, and evolutionary pressures [11, 22, 23]. Additionally, it facilitates the analysis of viral distribution and evolutionary dynamics across spatial and temporal scales, providing critical insights into the ecological and epidemiological patterns of MBVs.

Therefore, our study performed a comprehensive collection of mosquito samples across Guizhou Province during 2021 and 2022, followed by metagenomic sequencing of pooled samples from *Aedes*, *Anopheles*, *Armigeres*, and *Culex* mosquitoes. Bioinformatics analysis revealed the diversity and dynamic expression profiles of both known and novel viruses. Several significant zoonotic viruses were experimentally validated by cell isolation and reverse transcription-quantitative PCR (RT-qPCR). Additionally, gene sequencing provided valuable insights into the genetic evolution of JEV, Getah virus (GETV) and Banna virus (BAV). Comparative analysis further highlighted notable variations in viral diversity among mosquito species and elucidated differences in virome composition between Guizhou, other provinces in China, and other countries.

## Methods

### Mosquito sampling and preservation

In this study, we employed five different methods to capture wild mosquitoes: BG Mosquitaire CO_2_ traps (Bioagents, Germany), mosquito-killing lamps (Jixing Medical Technology Co., Ltd. Wuhan, China), manual collection, human bait traps, and oviposition traps. From July to September in 2021 and 2022, we conducted fieldwork across 22 locations in Guizhou Province, China, covering Zunyi city (Bozhou, Chishui, Tongzi), Tongren city (Dejiang, Jiangkou, Songtao), Qianxinan Buyei and Miao Autonomous Prefecture (Xingren, Zhenfeng, Anlong, Wangmo), Bijie city (Qixingguan, Qianxi, Weining), Qiannan Buyei and Miao Autonomous Prefecture (Cengdu, Libo, Luodian), Qiandongnan Miao and Dong Autonomous Prefecture (Liping, Rongjiang, Congjiang), Anshun city (Xixiu), and Liupanshui city (Zhongshan, Panzhou) (Supplementary: Table S1). The collected mosquitoes encompassed diverse ecological settings, including urban, suburban, rural, mountainous, and forested areas. All mosquito samples were placed in liquid nitrogen containers by species, collection site, and sampling year, then promptly transported to the laboratory and stored at − 80 °C.

### Vector-host confirmation

The captured mosquitoes were morphologically identified to species and genus level, followed by PBS buffer washing to remove contaminants. Cleaned mosquitoes were homogenized in sterile centrifuge tubes. Genomic DNA was extracted using the TIANamp Genomic DNA Kit (TIANGEN Biotech, Beijing, China). The cytochrome C oxidase subunit I (COI) gene was amplified by PCR using forward (5'-GGTCAACAAATCATAAAGATATTGG-3') and reverse (5'-TAAACTTCAGGGTGACCAAAAAATCA-3') primers. The PCR reaction mixture (25 µl total volume) consisted of 12.5 µl Dream Green PCR Master Mix, 2–5 µl DNA template, 1 µl each of forward and reverse primers, and RNase Free dH_2_O. PCR cycling conditions were: 94 °C for 1 min; 4 cycles of 94 °C for 40 s, 45 °C for 40 s, and 72 °C for 1 min; 29 cycles of 94 °C for 40 s, 51 °C for 40 s, and 72 °C for 1 min; and final extension at 72 °C for 5 min (TaKaRa, Beijing, China). PCR products were Sanger-sequenced by TsingKe Biotechnology Co., Ltd. (China) and species identification was confirmed via BLASTx (https://blast.ncbi.nlm.nih.gov/Blast.cgi?LINK_LOC=blasthome&PAGE_TYPE=BlastSearch&PROGRAM=blastx) searches against the NCBI nr database. Multiple sequence alignment was performed using MAFFT (v7.526) [24], with ambiguously aligned regions removed using trimAl (v1.4.1) [25]. The best-fit model was selected using ModelFinder in IQ-TREE (v2.3.4) [26] based on the Bayesian Information Criterion, and a maximum likelihood phylogenetic tree was constructed with 1000 bootstrap replicates.

### Virome library construction, sequencing, and assembly

Total RNA was isolated from mosquito tissues using TRIzol Reagent (Invitrogen, USA) according to the manufacturer’s protocol. The RNA was then purified using magnetic beads, converted to cDNA libraries, and prepared into sequencing libraries for subsequent analysis on the MGISEQ-2000 platform (MGI Tech Co., Ltd., Shenzhen, China). Raw sequencing data were quality-controlled using fastp (v0.23.2) [27]. Host sequences were removed through alignment to a custom database of target vector genera (*Anopheles*, *Armigeres*, *Culex*, and *Aedes*) using BWA-MEM (v0.7.17) [28] and SAMtools (v1.20) [29] (samtools view -f 4 -Su). rRNA sequences were eliminated by alignment against the SILVA rRNA database (v138.1, https://www.arb-silva.de/). Data quality was assessed using FastQC (v0.12.1) [30]. De novo assembly was performed using MEGAHIT (v1.2.9) [31] with optimized parameters (–k-min 21, –k-max 121, and –k-step 10). Finally, contigs from all libraries were merged and clustered using CD-HIT (v4.8.1) [32] (cd-hit-est -c 0.9 -n 10 -aS 0.9 -G 1c) to generate non-redundant sequences for downstream analyses.

### Detection of RNA-dependent RNA polymerase (RdRp) sequences

RdRp, a multifunctional enzyme crucial for RNA virus genome replication and transcription, is widely used to identify known and novel RNA viruses in metagenomic data. Assembled contigs were translated into protein sequences using EMBOSS (v6.6.0) [33] for RdRp annotation. Viral sequences were annotated via BLASTp searches against viral RdRp sequences using Diamond (v0.9.19) [34]. RdRp protein sequences of various viruses were obtained from Charon et al. [35] and downloaded from https://github.com/JustineCharon/RdRp-scan.

### Virus discovery

Three pipelines, Kraken (v2.1.3) [36], Kaiju (v1.10.1) [37] and MMseqs2 taxonomy [38], were sequentially used for taxonomic classification and viral contig identification. Kraken and Kaiju utilized custom virus databases, while MMseqs2 taxonomy used UniRef100. All classified contigs were integrated as potential viral sequences. Known viruses were identified using BLAST searches against three databases: ZVOER (the database of zoonotic and vector-borne viruses) [39], NCBI nr and viral genome nucleotide databases (downloaded June 22, 2024), with an E-value cutoff of 1e−5 and a maximum of one hit per sequence. Sequences confirmed in all three databases were classified as definitive viral hits, while those identified in two databases underwent manual validation based on RdRp protein conservation. Sequences with > 90% nucleotide or > 80% protein similarity to database entries were retained as known viruses. Unclassified sequences were subjected to novel virus prediction using geNomad (v1.8.0) [40], DeepVirFinder (v1.0) [41], and VirSorter2 [42], with consensus predictions retained. Finally, the longest representative sequence from each virus was selected for index construction and subsequent quantification using Salmon (v1.4.0) [43].

### Virus isolation

Mosquito samples were processed through a standardized virus isolation protocol. Following three PBS washes, pooled specimens were homogenized using a cryogenic grinder with stainless steel beads in RPMI-1640 medium (Gibco, Suzhou, China) supplemented with 2% fetal bovine serum (FBS). After centrifugation (12,000 × *g*, 20 min, 4 °C), supernatants were filtered through 0.22-μm membranes. For virus isolation, 100 μl filtrates were inoculated in duplicate into C6/36 (*Ae. albopictus*) and BHK-21 (baby hamster kidney) cell cultures. Following 1 h adsorption at respective optimal temperatures (28 °C for C6/36, 37 °C for BHK-21), cultures were maintained with 1 ml of 2% FBS medium for 6–7 days. Cultures exhibiting a cytopathic effect of over 75% were harvested, and the viral RNA was extracted for initial identification by RT-qPCR. This was followed by genomic analysis of the pathogenic isolates.

### Viral annotation and phylogenetic inference

For virus annotation, we used species classification from the “Virus discovery” section and identified the best ORF hit. ORFs were predicted using ORFfinder [44], followed by BLASTp searches against RdRp-scan database. RdRp sequences with ORFs > 200 amino acids were selected, and the longest overlapping sequences were retained for evolution analysis. Phylogenetic trees were constructed using the same pipeline as described in the COI analysis section, with PaPhy-ML [45] used for all inferences.

### Viral Shannon index and abundance diversity

Viral protein sequences from this study were combined with mosquito-related viral sequences from the RdRp-scan and ZOVER databases. Sequences were filtered, deduplicated, and clustered into Operational Taxonomic Units (OTUs) at 90% nucleotide similarity using VSEARCH (v2.28.1) [46]. For each sample, OTUs were quantified using Salmon (v1.4.0) [43] to obtain TPM expression, with values < 1 excluded to improve data quality before Shannon index calculation. The Shannon index of the virome was calculated using the following formula:

$$H=-\sum_{i=1}^{S} {p}_{i}\text{ln}({p}_{i}),$$

Here, $H$ represents the Shannon index, used to measure the diversity of OUTs within samples. $S$ denotes the total number of OTUs in a sample. ${p}_{i}$ = $\frac{{\text{TPM}}_{i}}{\sum_{j=1}^{S} {\text{TPM}}_{j}}$ is the relative abundance of the $i$-th OTU. Of which, ${\text{TPM}}_{i}$ is the TPM value of the $i$-th OTU, and $\sum_{j=1}^{S} {\text{TPM}}_{j}$ is the sum of TPM values for all OTUs in the sample.

## Results

### Overview of mosquito sample collection and virome data

**Fig. 1:**
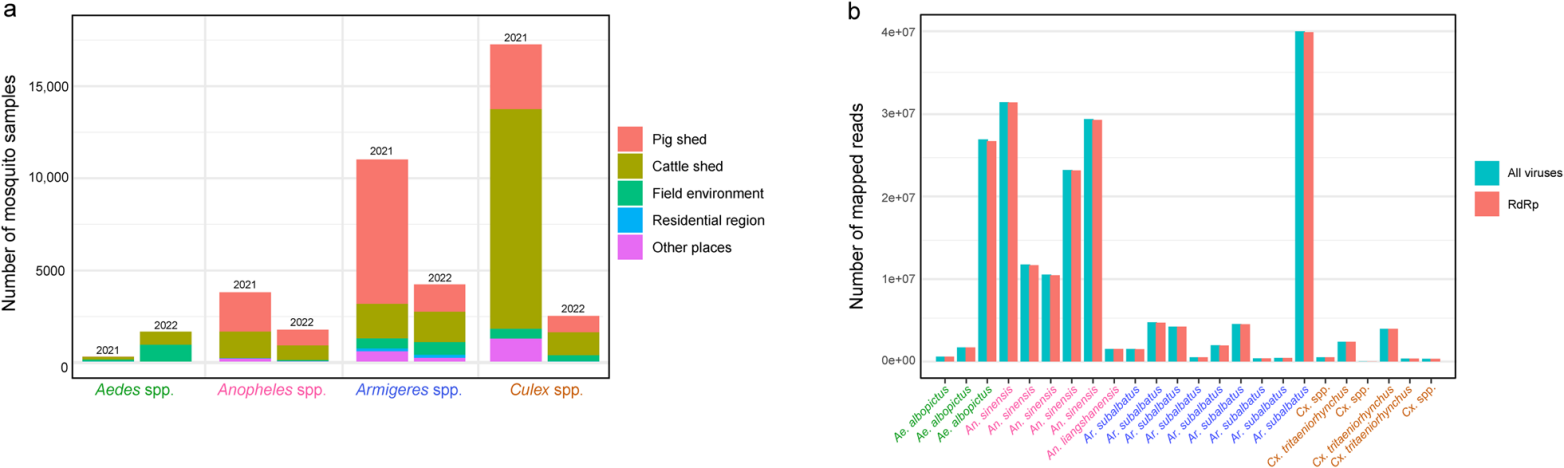
Overview of mosquito sampling and viral metagenomic sequencing. **a** Distribution of mosquito sampling habitats, including pig sheds, cattle sheds, field environments, residential areas, and other locations. **b** The number of sequencing reads mapped to NCBI reference viral genomes and viral RdRp sequences in the sequencing libraries

A total of 42,029 adult female mosquitoes were collected across eight autonomous prefectures or cities (LPS, BJ, AS, ZY, TR, QDN, QN, and QXN) in Guizhou Province (Supplementary: Table S1). Notably, the number of mosquitoes collected in QN and QXN each exceeded 10,000 individuals. To ensure high reliability in species identification, we combined morphological examination using an ultra-depth-of-field microscope with PCR-based molecular diagnostics targeting the mitochondrial COI gene. In total, we identified 11 mosquito species across 2 subfamilies and 4 genera. These included *Anopheles sinensis*, *An. liangshanensis*, and the *An. baileyi* complex within the genus *Anopheles*; *Culex tritaeniorhynchus*, *Cx. quinquefasciatus*, *Cx. theileri*, *Cx. orientalis*, and *Cx. mimeticus* within the genus *Culex*; *Aedes albopictus* and *Ae. vexans* within the genus *Aedes*; and *Armigeres subalbatus* within the genus *Armigeres*. Notably, the *An. baileyi* complex represents a newly recorded mosquito species in Guizhou Province. Sampling locations included pig sheds, cattle sheds, fields environment, and residential areas. Specifically, mosquitoes from the genera *Anopheles*, *Armigeres*, and *Culex* were primarily collected from pig shed and cattle shed, while those from the genus *Aedes* were predominantly found in field environment and residential areas (Fig. 1a).

A total of 3,860,677,506 reads were generated, averaging 160,861,563 reads per pool. After removing host-derived reads, 208,201,790 reads remained, corresponding to an average of 8,675,075 reads per pool. Of these, 203,145,589 reads were successfully mapped to viral reference genomes in the NCBI database, averaging 8,464,400 reads per pool, while de novo assembly generated 51,659 contigs, averaging 2152 contigs per pool (Supplementary: Table S2). Given that RNA viruses typically encode RdRp, we assessed the enrichment of reads mapping to RdRp sequences. A total of 202,349,095 reads were mapped, accounting for 99.61% of the total viral reads, demonstrating the high sensitivity of virus detection enabled by the depth sequencing data in this study (Fig. 1b and supplementary: Table S2). To analyze viral composition, we classified and annotated sequences using Kaiju, Kraken2, and MMseqs2 (Supplementary: Table S3). Additionally, we performed BLAST searches against the nr database, NCBI Virus Genome, ZVOER, and RdRp-Scan databases, followed by a comparative analysis and curation of these sequences (Supplementary: Table S4). This integrative approach facilitated the identification of potentially known viruses and successfully detected several significant zoonotic viruses, including JEV, GETV and BAV.

### Taxonomic assignments of mosquito-derived viruses

**Fig. 2:**
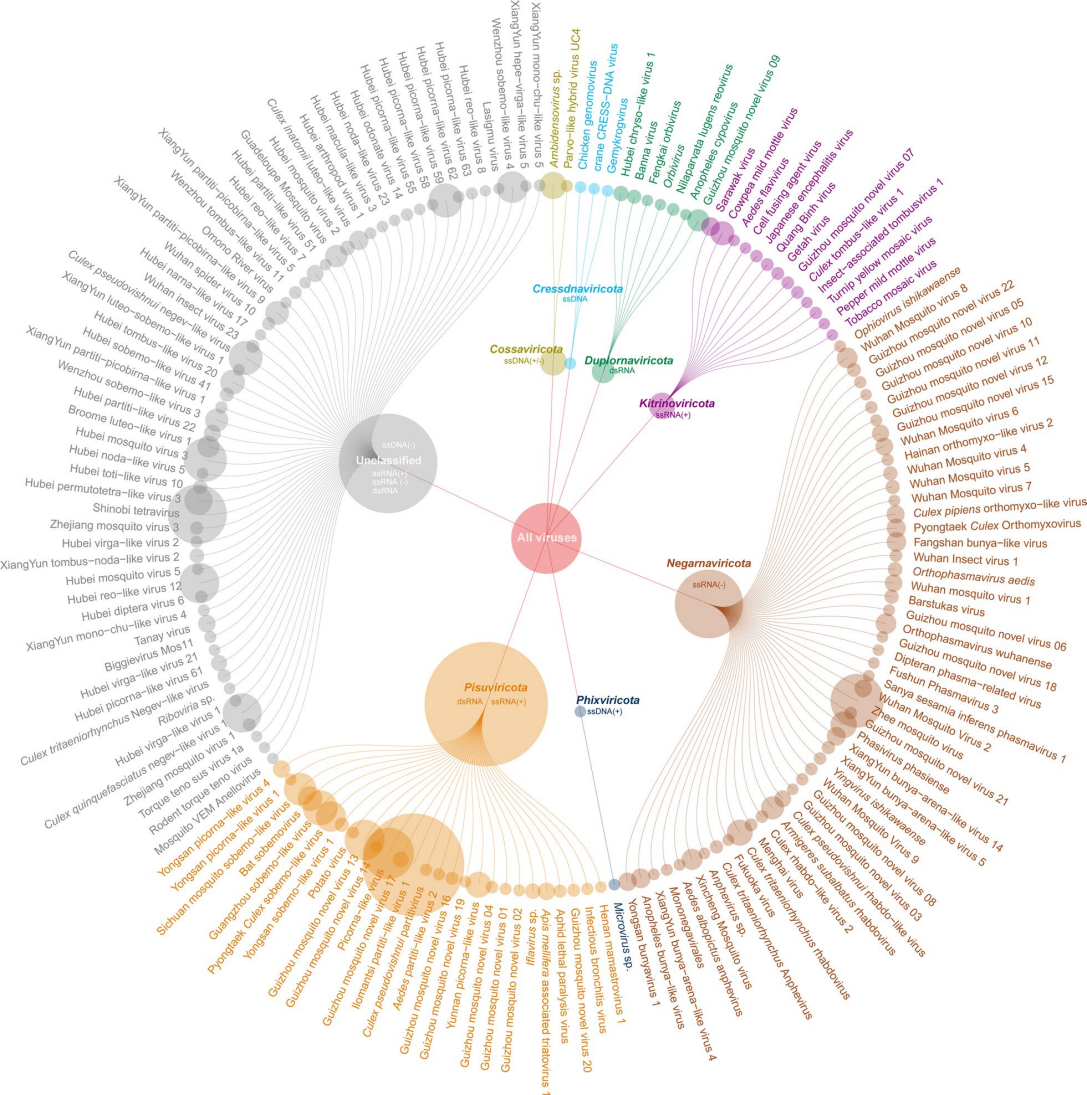
The relationships between the known viruses and previously uncharacterized viruses identified in this study and their corresponding phyla

Based on the results generated by the virus annotation and classification pipeline (see Methods section), we conducted a comprehensive analysis to determine the taxonomic assignments of both known and previously uncharacterized viruses. In this viral metagenomic survey across Guizhou Province, a total of 162 viral fragments were identified, comprising 140 known viruses and 22 previously uncharacterized viruses (Fig. 2 and supplementary: Table S5). Among these, 103 viruses were classified into known phyla, including *Cossaviricota*, *Cressdnaviricota*, *Duplornaviricota*, *Kitrinoviricota*, *Negarnaviricota*, *Phixviricota*, and *Pisuviricota*, while 59 viruses could not be assigned to any known phyla. Further analysis revealed that 96 viruses were allocated to 32 known viral families, with dominant families such as *Phasmaviridae* (10.4%), *Rhabdoviridae* (9.3%), *Orthomyxoviridae* (8.3%), and *Iflaviridae* (7.2%). Additionally, five viruses were predicted to belong to newly identified viral families, while 61 viruses remained unclassified at the family level.

### Comparative analysis of viral distribution

We conducted a comprehensive comparative analysis of viral species distribution across four mosquito genera (*Aedes*, *Anopheles*, *Armigeres*, and *Culex*) at the phylum, family, and species levels. Using Venn diagram analysis, we identified overlaps and unique associations within the viral communities. At the phylum level, three phyla, *Negarnaviricota*, *Kitrinoviricota*, and *Pisuviricota*, were shared among all mosquito pools. Moreover, two phyla, *Cossaviricota* and *Duplornaviricota*, were exclusively shared among the genera *Anopheles*, *Armigeres*, and *Culex*, while *Cressdnaviricota* and *Phixviricota* were uniquely associated with *Anopheles* (Supplementary: Fig. S1a). At the family level, five viral families, *Parvoviridae*, *Phasmaviridae*, *Phenuiviridae*, *Solemoviridae*, and *Xinmoviridae*, were shared across all mosquito pools, with varying degrees of overlap among different mosquito genera (Supplementary: Fig. S1b). Notably, several viral families with significant public health implications, such as *Coronaviridae*, *Flaviviridae*, and *Togaviridae*, were identified. At the species level, only three viruses, Sichuan mosquito sobemo-like virus, Guangzhou sobemo-like virus, and Zhee mosquito virus, were shared across all four mosquito pools, while other viruses exhibited variable distribution patterns among different genera (Supplementary: Fig. S1c). Below, we present a detailed analysis of viral families and species with significant public health implications.

Viruses belonging to the *Coronaviridae* family were identified, including infectious bronchitis virus (IBV), a γ-coronavirus [47] detected in mosquito genera *Anopheles*, *Armigeres*, and *Culex*. Within the *Flaviviridae* family, virus sequences were detected in *Aedes*, *Anopheles*, and *Culex*, but not in *Armigeres*. Quang Binh virus (QBV) belonging to the *Flaviviridae* family, an ISV, was also identified and confirmed by viral screening and whole-genome sequencing, as previously reported by us [48]. Although no pathogenic flaviviruses such as DENV, CHIKV, ZIKV, or WNV were detected, sequences corresponding to JEV were present in samples from multiple sampling sites. Experimental validation demonstrated that JEV can induce cytopathic effects in C6/36 cells, which was further confirmed by RT-qPCR analysis (Supplementary: Fig. S2). Additionally, GETV, a member of *Togaviridae* family, was detected in *Aedes* and *Armigeres* mosquitoes. Its presence was validated by observing cytopathic effect in C6/36 cells and confirmed by RT-qPCR analysis (Supplementary: Fig. S3).

### Genotype analysis of zoonotic viruses (JEV, JETV and BAV)

**Fig. 3:**
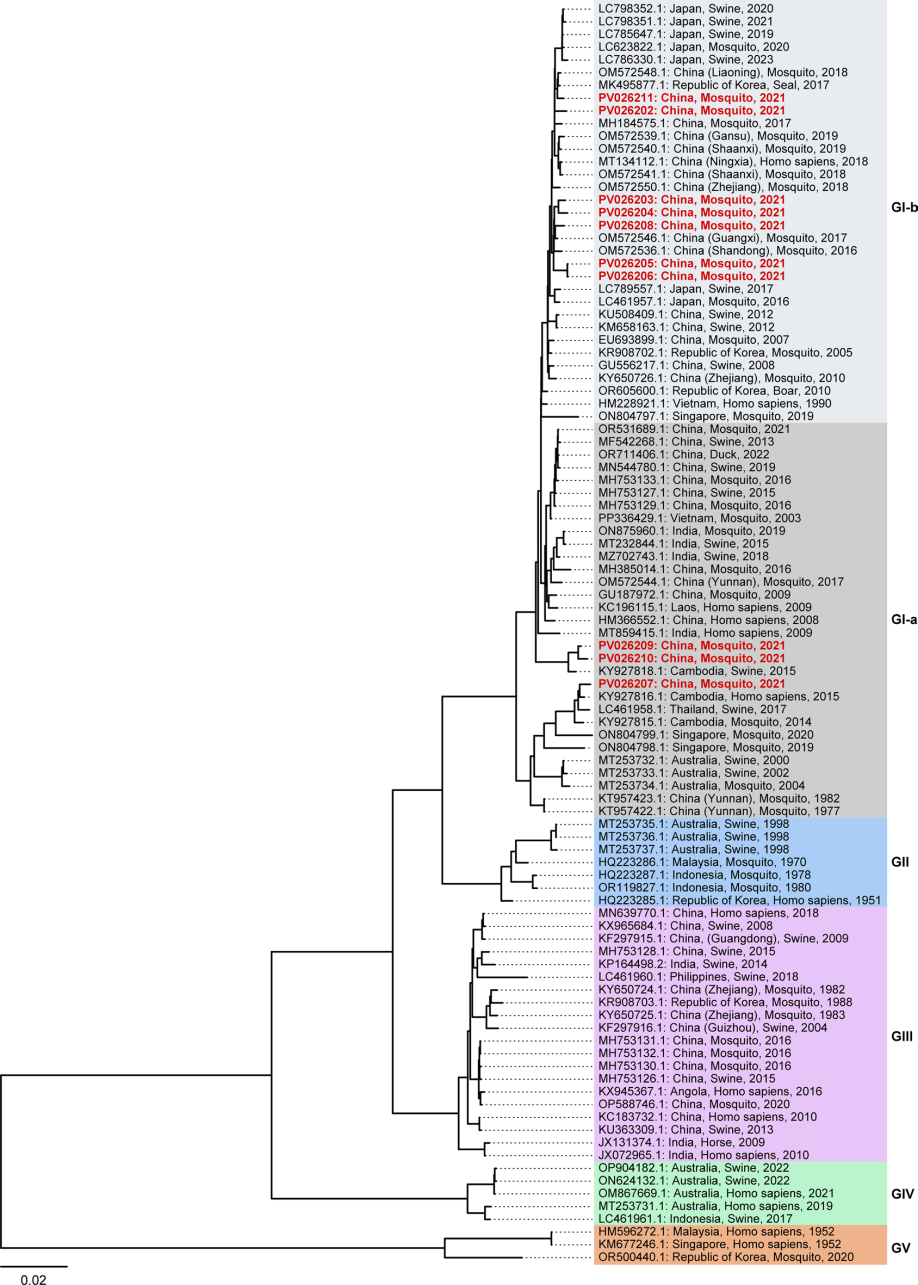
Maximum likelihood phylogenetic tree of JEV. The tree was constructed using IQ-TREE software based on complete genome sequences, applying the best-fit model (GTR + F + I + G4). Branch lengths are indicated by the scale bar (length = 0.02). Root-to-tip genetic distances were analyzed against sampling dates using TempEst to assess temporal signals and validate the molecular clock model for evolutionary analyses. Under this model, JEV strains are classified into five genotypes: GI (further subdivided into GI-a and GI-b), GII, GIII, GIV, and GV. Sequences identified in this study are highlighted in red

JEV was detected in ten mosquito pools and further confirmed by viral isolation in cell culture followed by whole-genome sequencing. Of these, two pools (GenBank ID: PV026202 and PV026203) were collected from Tongren (TR), three (GenBank ID: PV026205, PV026206, and PV026208) from Zunyi (ZY), and five (GenBank ID: PV026204, PV026207, PV026209, PV026210, and PV026211) from Qianxinan (QXN). To investigate the genetic diversity and temporal dynamics of these JEV strains, we retrieved 89 complete JEV genome sequences from GenBank and combined them with the ten newly sequenced genomes from this study to construct a comprehensive phylogenetic tree. Temporal signal analysis confirmed that all JEV samples belonged to Genotype I (GI). Among them, three isolations (GenBank ID: PV026207, PV026209 and PV026210) were assigned to the GI-a subgenotype, while the remaining seven (GenBank ID: PV026202, PV026203, PV026204, PV026205, PV026206, PV026208, and PV026211) were classified as GI-b (Fig. 3). Notably, all five strains (GenBank ID: PV026202, PV026203, PV026205, PV026206, and PV026208) from TR and ZY belonged to GI-b. In contrast, three (GenBank ID: PV026207, PV026209, and PV026210) of the five isolations from QXN fell within the GI-a subgenotype, with the exception of PV026204 and PV026211, which were grouped into GI-b. Geographically, TR and ZY are located in northern Guizhou, whereas QXN lies in the southern region (Supplementary: Table S1), suggesting a latitudinal gradient in JEV genotype distribution, with GI-b strains potentially more prevalent in northern regions.

**Fig. 4:**
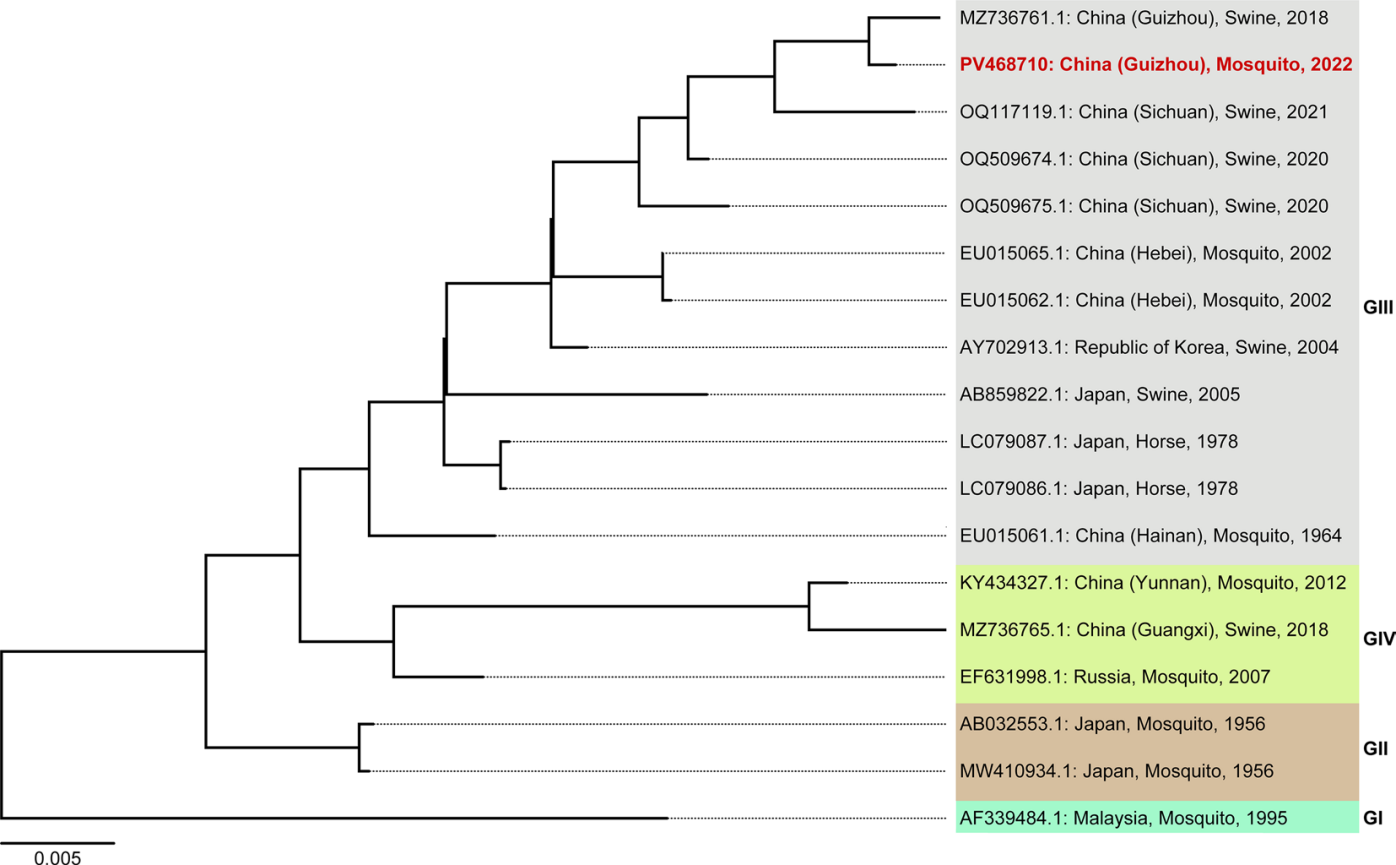
Maximum likelihood phylogenetic tree of GETV based on E2 gene. The tree was constructed using IQ-TREE with the best-fit model (GTR + F + G4). Branch lengths are indicated by the scale bar (length = 0.005). Root-to-tip genetic distances were analyzed against sampling dates using TempEst to assess temporal signals and validate the molecular clock model for evolutionary analyses. Under this model, GETV strains are classified into four groups: G1, G2, G3 and G4. The E2 gene sequence identified in this study is highlighted in red

GETV was detected in *Ar. subalbatus* from TR, and its genotype was confirmed through cell isolation and Sanger sequencing. The E2 gene, regarded as the gold standard for GETV genotyping, enables classification into four phylogenetic groups (GI–GIV) worldwide. As shown in Fig. 4, phylogenetic analysis revealed that the GETV strain (GenBank ID: PV468710) identified in this study belongs to the GIII strain and is closely related to the strain detected in swine in Guizhou in 2018 (GenBank ID: MZ736761.1). To date, GI and GII strains have not been reported in China, while GIV strain was previously detected in Yunnan in 2012 (GenBank ID: KY434327.1) and Guangxi in 2018 (GenBank ID: MZ736765.1).

**Fig. 5:**
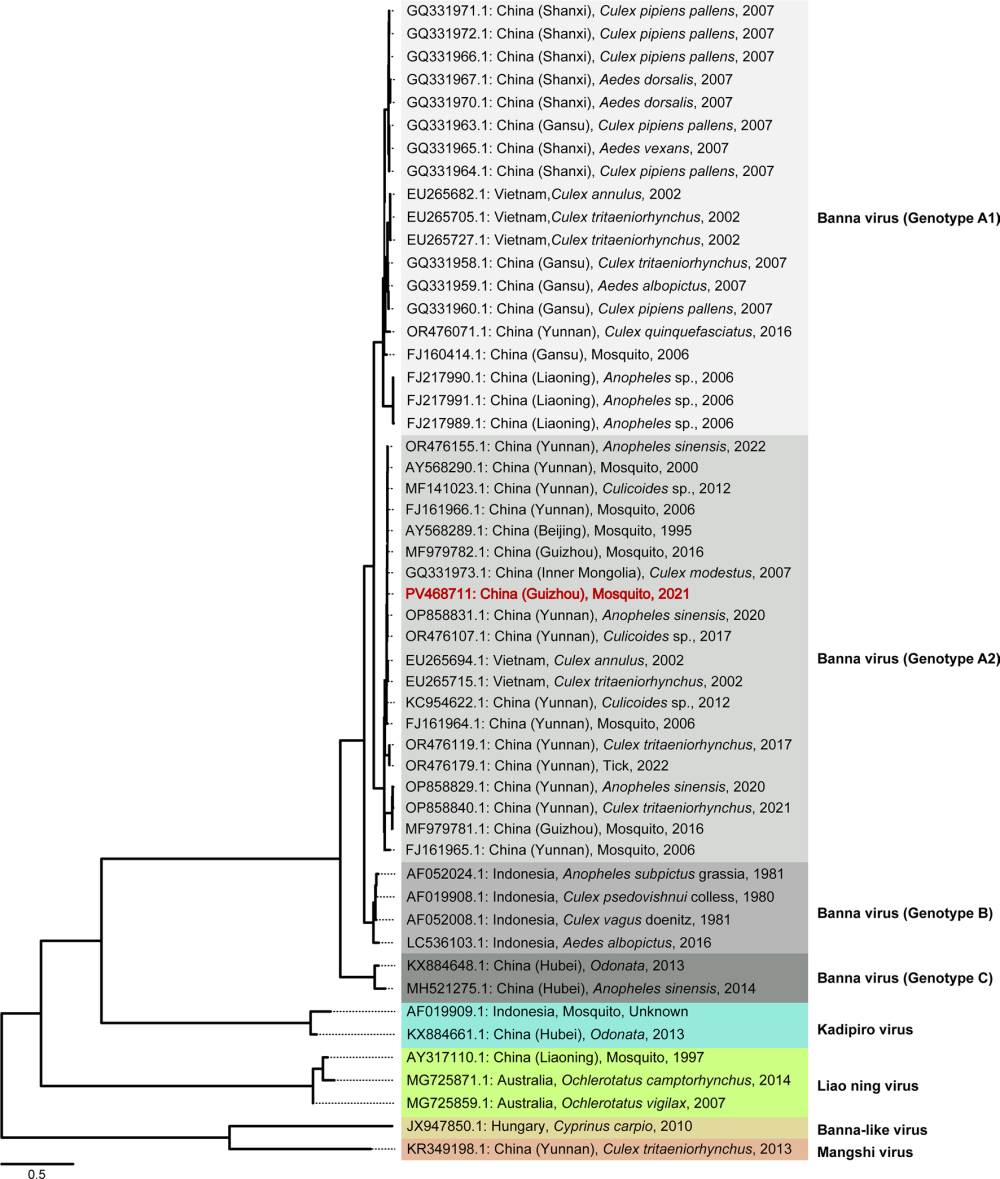
Maximum likelihood phylogenetic tree of BAV based on VP12 gene. The tree was constructed based on the VP12 gene using IQ-TREE with the best-fit model (HKY + F + R2). Branch lengths are indicated by the scale bar (length = 0.5). Root-to-tip genetic distances were analyzed against sampling dates using TempEst to assess temporal signals and validate the molecular clock model for evolutionary analyses. The inter-species evolutionary analysis revealed that the virus belongs to the BAV clade, which is classified into three subtypes: **A** (further divided into subtypes A1 and A2), **B**, and **C**. The VP12 gene sequence identified in this study is highlighted in red

Finally, BAV, a representative segmented RNA virus, was primarily detected in *Cx. quinquefasciatus* collected from QXN. The BAV genome comprises 12 segments (VP1–VP12), among which the VP12 gene serves as a principal marker for subtype classification. Phylogenetic analysis placed the identified virus within the BAV clade of 12-segmented RNA viruses, exhibiting clear genetic divergence from related viruses such as Kadipiro virus and Liao ning virus (Fig. 5). Based on VP12 gene sequences, the Guizhou strain characterized in this study (GenBank ID: PV468711) was assigned to genotype A2, clustering with two previously reported A2 genotype strains from Guizhou in 2016. The newly characterized strain showed a closer genetic relationship to one of these earlier strains (GenBank ID: MF979782.1), while being more distantly related to the other (GenBank ID: MF979781.1), indicating the presence of distinct evolutionary lineages and transmission histories of BAV within the Guizhou region.

### Phylogenetic analysis and expression profiling of long-chain RdRp proteins

The RdRp gene serves as a universally conserved marker across RNA viruses, facilitating phylogenetic classification and evolutionary studies. In this study, we selected RdRp sequences with lengths ≥ 200 amino acids from 34 previously characterized viruses and 22 newly identified viruses for phylogenetic classification and transcriptomic profiling. These viruses span four phyla (*Duplornaviricota*, *Kitrinoviricota*, *Negarnaviricota*, and *Pisuviricota*) and encompass 15 distinct families (*Totiviridae*, *Spinareoviridae*, *Tombusviridae*, *Togaviridae*, *Phasmaviridae*, *Peribunyaviridae*, *Phenuiviridae*, *Rhabdoviridae*, *Xinmoviridae*, *Orthomyxoviridae*, *Iflaviridae*, *Dicistroviridae*, *Solemoviridae*, *Partitiviridae*, and *Picornaviridae*).

**Fig. 6:**
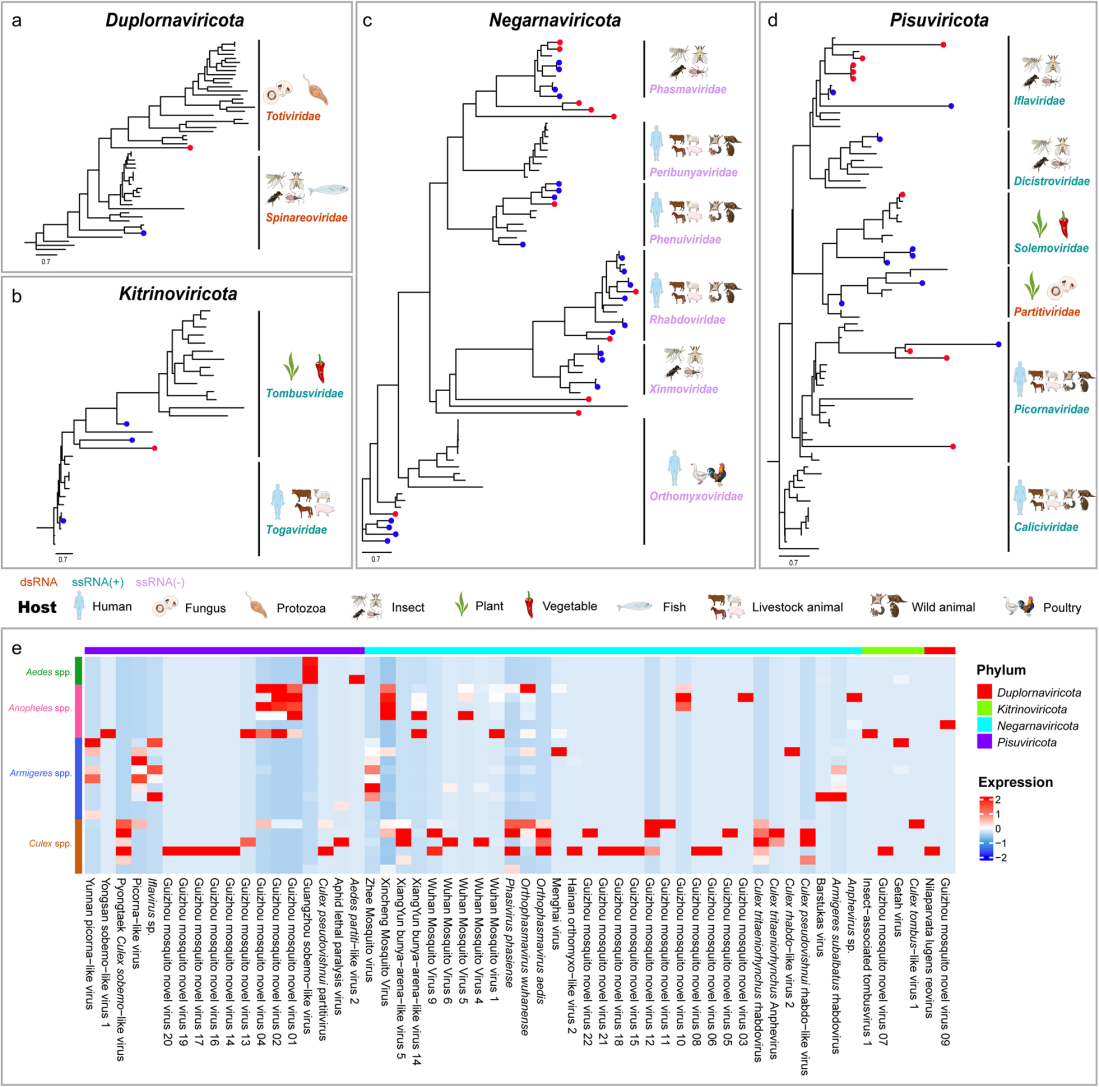
Maximum likelihood phylogenetic trees based on RNA-dependent RNA polymerase (RdRp) sequences (≥ 200 amino acids in length) depict viruses within the phyla *Duplornaviricota* (**a**), *Kitrinoviricota* (**b**), *Negarnaviricota* (**c**), and *Pisuviricota* (**d**). Branch lengths are represented by the scale bar (length = 0.7). The figure highlights the primary host sources for each viral family, including humans, fungi, protozoa, insects, plants, vegetables, fish, livestock animals, wildlife, and poultry. Different RNA virus types are color-coded: dsRNA, ssRNA (+), and ssRNA (−). **e** A heatmap illustrates the expression profiles of viruses with RdRp protein sequences ≥ 200 amino acids across various mosquito species, with red indicating high expression levels and blue indicating low expression levels

Within the phylum *Duplornaviricota*, two viruses were identified based on RdRp sequences: the previously known Nilaparvata lugens reovirus (NLRV) and a newly discovered virus, Guizhou mosquito novel virus 9 (GMNV9; GenBank ID: PV468720). NLRV, a dsRNA virus, is closely associated with the brown planthopper (*Nilaparvata lugens*), a significant agricultural pest [49]. Although NLRV typically exhibits no significant pathogenicity to its host, it may interact synergistically with other viruses, potentially influencing host reproductive or behavior. Phylogenetic analysis places NLRV within the family *Spinareoviridae*, known for infecting a broad range of hosts and are primarily transmitted via insect vectors (Fig. 6a and supplementary: Fig. S4). Notably, NLRV has been officially recognized by the International Committee on Taxonomy of Viruses (ICTV) as an ISV [50]. The newly identified virus, GMNV9 belongs to the family *Totiviridae* and shares a close genetic relationship with Camponotus nipponicus virus (CNV), forming a distinct monophyletic clade (Fig. 6a and supplementary: Fig. S4). CNV is associated with ants (*Formicidae*) and may influence colony behavior or health [51]. By inference, GMNV9 is also considered an ISV with potential ecological functions. In addition, expression profiling revealed high expression levels of NLRV and GMNV9 in *Culex* and *Anopheles* mosquitoes, respectively (Fig. 6e).

In the phylum *Kitrinoviricota*, three known viruses, namely Insect associated tombusvirus 1 (IAT1), *Culex tombus*-like virus 1 (CTLV1), and GETV, were identified, along with a novel virus designated GMNV7 (GenBank ID: PV468718) (Fig. 6b and supplementary: Fig. S5). IAT1 and CTLV1 are representative ISVs classified as ssRNA (+) viruses within the family *Tombusviridae*. Although *Tombusviridae* predominantly comprises plant-associated viruses [52], the discovery of IAT1, CTLV1, and GMNV7 suggests potential ecological interplay involving insects and plants, possibly influencing plant–insect interactions. In contrast, the family *Togaviridae*, also consisting of ssRNA (+) viruses, includes GETV, a significant zoonotic pathogen. *Togaviridae* members are recognized for their roles in zoonotic and public health concerns, with mosquito-borne Alphaviruses (e.g., CHIKV) being globally distributed and of substantial clinical importance. The identification of GETV underscores the critical role of *Togaviridae* in zoonotic disease transmission and public health surveillance.

The phylum *Negarnaviricota* encompasses a diverse array of ssRNA (-) viruses, including insect-specific families (*Phasmaviridae* and *Xinmoviridae*) and families capable of infecting humans or animals (*Peribunyaviridae*, *Phenuiviridae*, *Rhabdoviridae*, and *Orthomyxoviridae*) (Fig. 6c and supplementary: Fig. S6). Among ISVs, seven known viruses and two novels were discovered. Additionally, three unclassified viruses, namely GMNV5 (GenBank ID: PV468716), GMNV11 (GenBank ID: PV468722), and GMNV22 (GenBank ID: PV468733), exhibited close genetic relationships with the *Phasmaviridae* family, while GMNV10 (GenBank ID: PV468721) and GMNV12 (GenBank ID: PV468723) were related to the *Xinmoviridae* family, suggesting the emergence of new evolutionary lineages. Regarding viruses infecting humans or animals, ten known viruses and four newly evolved ones were identified, distributed across the *Phenuiviridae*, *Rhabdoviridae*, and *Orthomyxoviridae* families. Notably, *Phenuiviridae* viruses have zoonotic potential [53], *Rhabdoviridae* includes lethal human pathogens like rabies virus [54], and *Orthomyxoviridae* contains influenza viruses with significant public health and agricultural impacts [55]. These viruses were predominantly highly expressed in *Culex* mosquitoes (Fig. 6e), yet the classification of many remains ambiguous.

In the phylum *Pisuviricota*, four viral families, namely *Iflaviridae*, *Dicistroviridae*, *Solemoviridae*, and *Partitiviridae*, were identified, none of which infect humans or animals (Fig. 6d and supplementary: Fig. S7). *Iflaviridae*, *Dicistroviridae*, and *Solemoviridae* belong to the ssRNA (+) virus category, while *Partitiviridae* is a dsRNA virus family. *Iflaviridae* and *Dicistroviridae* are primarily insect-specific [56, 57], whereas *Solemoviridae* predominantly infect plants and rely on insect vectors for transmission [58]. *Partitiviridae* are broadly distributed across fungi, plants, and protists [59]. Our analysis revealed three known and six newly evolved ISVs, as well as three plant-associated viruses within these families. Among all identified viral families, only *Picornaviridae*, a significant ssRNA (+) family capable of infecting humans and animals, was observed (Fig. 6d and supplementary: Fig. S7). This family includes Picorna-like virus and three newly evolved viruses, namely GMNV13 (GenBank ID: PV468724), GMNV14 (GenBank ID: PV468725), and GMNV17 (GenBank ID: PV468728).

### Alpha diversity of virus OTUs among different mosquito species

**Fig. 7:**
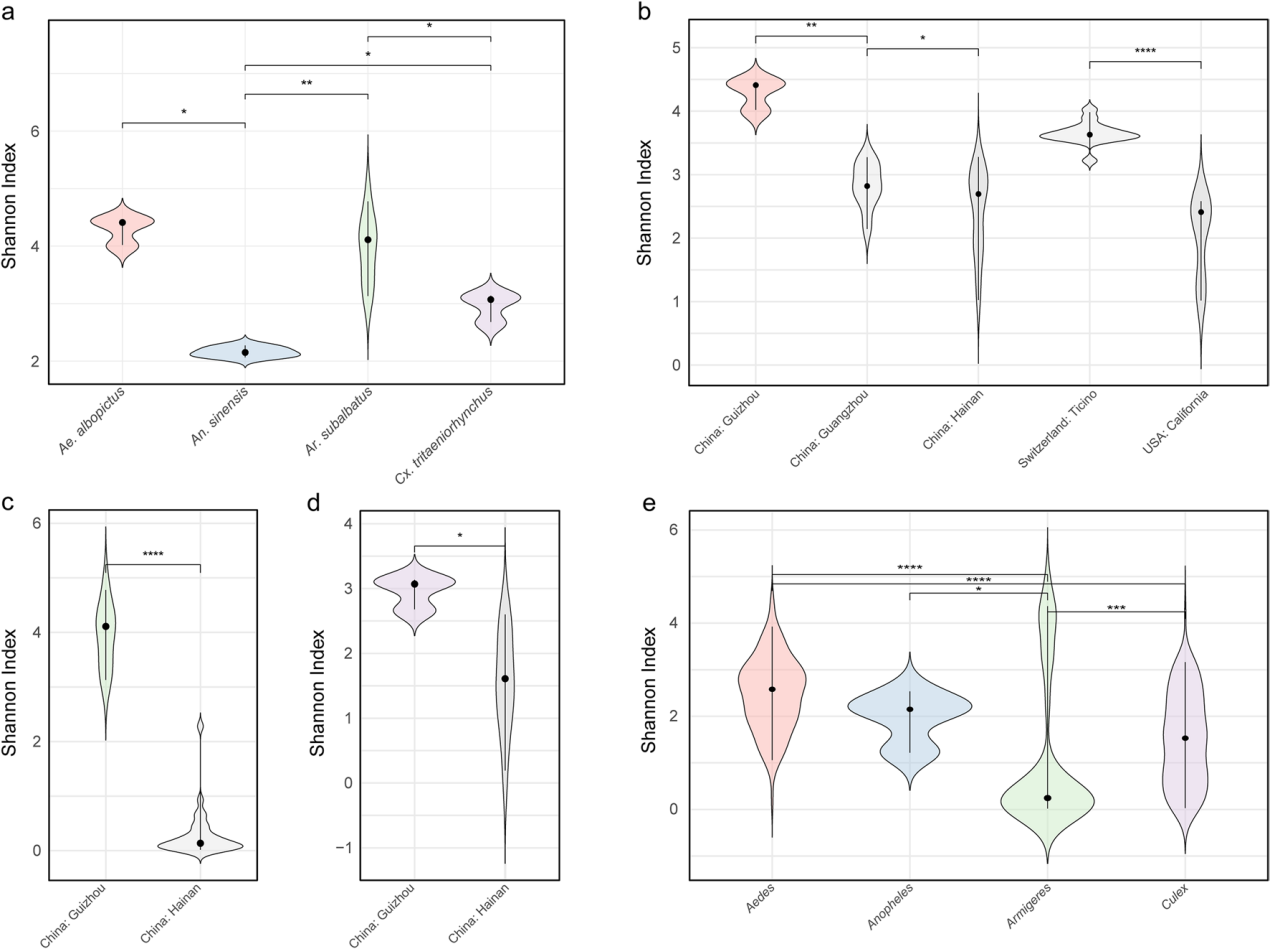
Alpha diversity of virus OTUs across mosquito species. **a** Comparison among the four most abundant mosquito species collected in this study: *Ae. albopictus, An. sinensis, Ar. subalbatus,* and *Cx. tritaeniorhynchus*. **b** Diversity of *Ae. albopictus* from Guizhou Province compared to Guangzhou (China), Hainan (China), Ticino (Switzerland), and California (USA). **c** Diversity of *Ar. subalbatus* from Guizhou compared with Guangzhou and Hainan. **d** Diversity of *Cx. tritaeniorhynchus* from Guizhou compared with Guangzhou and Hainan. **e** Global comparison of diversity across four mosquito genera: *Aedes*, *Anopheles*, *Armigeres*, and *Culex*. Statistical significance was determined using the Wilcoxon test, with significance levels indicated as *P* < 0.05 (*), *P* < 0.01 (**), *P* < 0.001 (***), and *P* < 0.0001 (****)

In the mosquito sample survey, *Cx. tritaeniorhynchus* and *Ar. subalbatus* were identified as the most prevalent mosquito species in Guizhou, followed by *An. sinensis* and *Ae. albopictus*. To evaluate the differences among mosquito species, we calculated the Shannon index for each viral library. The Shannon index considers both species abundance and evenness, with higher values indicating greater community complexity. Among the surveyed mosquito species, a comparison of the alpha diversity of virus OTUs revealed that, *Ae. albopictus* exhibited the highest diversity level, followed by *Ar. subalbatus* and *Cx. tritaeniorhynchus*, while *An. sinensis* showed the lowest diversity level (Fig. 7a). To investigate geographic differences among mosquito species, we compared the diversity levels of *Ae. albopictus*, *Ar. subalbatus*, and *Cx. tritaeniorhynchus* in Guizhou with those sequenced from other regions. The results showed that *Ae. albopictus* in Guizhou had the highest diversity levels compared to those in Guangzhou (China), Hainan (China), Ticino (Switzerland), and California (USA) (Fig. 7b), suggesting a more complex viral community in *Ae. albopictus* from Guizhou. For *Ar. subalbatus*, the diversity level in Guizhou was significantly higher than that in Hainan (*P* ≤ 0.0001) (Fig. 7c). Similarly, for *Cx. tritaeniorhynchus*, Guizhou showed significantly higher diversity levels than Hainan (Wilcoxon test: 0.01 < *P* ≤ 0.05) (Fig. 7d). Finally, we also compared 325 viromes from 10 other countries or regions, revealing that *Aedes* mosquitoes had the highest alpha diversity of viral OTUs overall, followed by *Anopheles* and *Culex*, while *Armigeres* exhibited the lowest diversity level (Fig. 7e and supplementary: Table S6). These findings highlight significant differences in OTU alpha diversity across different mosquito species.

## Discussion

The unique karst topography of Guizhou provides an exceptional natural laboratory for studying mosquito distribution, ecological diversity, and disease transmission. This distinctive landscape offers valuable insights into vector-borne diseases, particularly in understudied ecological settings. In this study, we collected over 40,000 adult mosquitoes from diverse locations across nearly the entire province, representing 11 mosquito species. Notably, the *An. baileyi* complex was recorded in Guizhou, indicating a significant geographical expansion beyond its previously reported ranges in Bhutan, Thailand, and Yunnan Province of China [60, 61]. However, the migratory routes and ecological roles of *An. baileyi* remain poorly understood, necessitating further ecological and genetic investigations to clarify its potential role in disease transmission and local biodiversity.

Our analyses revealed that most viromes are conserved within the same mosquito species across different locations and years, particularly in *Ae. albopictus, An. sinensis, Ar. subalbatus,* and *Cx. tritaeniorhynchus*, which are also major virus carriers in other Chinese provincial-level administrative divisions (PLADs), such as Shaanxi, Gansu, Ningxia, Shandong, and Yunnan [19, 21, 62]. This persistence suggests a stable host-virus association, potentially influenced by host-specific ecological factors, viral evolutionary pressures, or vector-virus interactions. Interestingly, our comparative study identified significant differences in virome composition, expression profiles, and genetic diversity among these dominant mosquito species. These findings underscore the importance of further investigations into the ecological and genetic factors driving these variations to better understand mosquito-virus dynamics and inform targeted vector control strategies.

The intricate relationship between arboviruses and their human and animal hosts remains a pivotal area of research, particularly due to the risks of cross-species transmission. In our study, we identified a diverse array of known and novel mosquito-borne viruses from the families *Flaviviridae*, *Orthomyxoviridae*, *Sedoreoviridae*, and *Togaviridae*, which carry significant implications for public health. Within the *Flaviviridae* family, we identified several members of the genus *Orthoflavivirus*, including Aedes flavivirus (AEFV), Cell fusing agent virus (CFAV), QBV, and JEV. Of which, AEFV, CFAV, and QBV are insect-specific flaviviruses known for vertical transmission and potential interference with pathogenic flavivirus infections [48, 63], while JEV remains a major public health concern. These ISVs warrant further investigation to elucidate their biology and potential roles in disease control. Similarly, within the *Orthomyxoviridae* family, several viruses have been reported in China, including Wuhan mosquito viruses (types 4–7), Hainan orthomyxo-like virus 2, as well as the newly identified GMNV15 (GenBank ID: PV468726) in our study. However, their taxonomic classification and biological characteristics are still poorly understood, necessitating further research.

We also observed significant findings within the *Sedoreoviridae* and *Togaviridae* families. In the *Sedoreoviridae* family, segmented viruses such as Fengkai orbivirus (FKOV) and BAV were identified. FKOV, initially discovered in Guangdong Province of China, has potential as a severe human pathogen [64], while BAV, with its broad host range including pigs, cattle, ticks, mosquitoes, and humans, underscores its zoonotic potential, particularly as an encephalitic pathogen [65]. These findings highlight the urgent need for continuous monitoring of these viruses to prevent potential outbreaks. Within the *Togaviridae* family, GETV was detected, which affects multiple vertebrate species, including humans, monkeys, birds, pigs, and horses. The increasing prevalence of GETV across China underscores the need for in-depth epidemiological and clinical studies to better understand its public health impact [66, 67]. Collectively, these findings emphasize the importance of exploring the transmission dynamics, host-pathogen interactions, and evolutionary trajectories of these viruses to mitigate emerging health risks.

JEV remains endemic in Asia and the Western Pacific, posing a significant burden, particularly on children in endemic regions and travelers of all ages [68]. Vaccination remains the only sustainable preventive strategy due to the lack of effective antiviral treatments. JEV is classified into five genotypes (GI to GV), with GI recently replacing GIII as the dominant genotype in Asia [69]. However, current commonly used vaccines, including the SA14-14-2 live-attenuated and Nakayama inactivated vaccines, are based on GIII strains [70]. While JEV genotypes belong to a single serotype, significant antigenic variation exists, with at least five distinct antigenic groups identified [71–73]. This variation raises concerns about the efficacy of GIII-based vaccines against GI strains, whose dominance may be driven by enhanced transmissibility, host adaptation, or antibody escape. Our findings show that the majority of JEVs belong GI, therefore highlighting the urgent need for next-generation vaccines that address ongoing antigenic and genotypic shifts.

Despite its comprehensive scope, this study has some limitations. Our focus on known viruses limited the exploration of novel viral characteristics and dynamics, partly due to reliance on predictive bioinformatics tools. Furthermore, aggregating mosquitoes from predominantly common species (*Ae. albopictus, An. sinensis, Ar. subalbatus,* and *Cx. tritaeniorhynchus*) resulted in insufficient nucleic acid for constructing virome libraries for rarer species. The lack of reference genomes for species like *An. mon* and *An. lia* further complicated the identification of virome library reads, particularly for shared endogenous fragments. Future studies should prioritize the cultivation and sequencing of newly identified viruses, habitat mapping, and genome sequencing of rare mosquito species to enhance the understanding of their virome profiles. These efforts will provide more accurate baseline data on mosquito-borne viruses in Guizhou and southwestern China, strengthening public health knowledge and response strategies. Furthermore, the application of long-read sequencing technologies facilitates the assembly of more complete viral genomes, providing deeper insights into viral diversity and evolution.

## Conclusions

This study systematically characterized the viral metagenomic profiles of mosquito viruses across Guizhou Province through the analysis of over 40,000 mosquito samples. Our findings demonstrate that the complex ecological environment provides ideal conditions for mosquito propagation and survival, with the *An. baileyi* complex recorded in Guizhou. We observed *Ae. albopictus, An. sinensis, Ar. subalbatus,* and *Cx. tritaeniorhynchus* as dominant mosquito species in the region. Viral presence was detected in both single and multiple mosquito genera, indicating that viral transmission is not limited to specific vectors. Notably, zoonotic viruses of significant public health concern, including JEV, GETV, and BAV, were identified, underscoring the urgent need to strengthen vector-borne virus surveillance and early warning systems. Moreover, metagenomic sequencing revealed significant differences in viral community enrichment and diversity among mosquito species. *Ae. albopictus* exhibited the highest viral diversity, followed by *Ar. subalbatus* and *Cx. tritaeniorhynchus*, while *An. sinensis* showed the lowest diversity. Compared to viral metagenomic data from other PLADs in China, *Ae. albopictus*, *Ar. subalbatus*, and *Cx. tritaeniorhynchus* in Guizhou exhibited significantly higher viral diversity, further highlighting the distinct ecological characteristics of mosquito viromes in this region.

## Supplementary Information

Additional file 1. Fig. S1 Venn diagrams showing the overlap of viruses identified at the phylum a, family b, and species c levels. Numbers indicate the counts of intersecting or unique phyla, families, and species. In panel c, red-labeled viruses (Getah virus, Japanese encephalitis virus, and Quang Binh virus) represent species validated through cell isolation and gene sequencing

Additional file 2. Fig. S2 JEV Cell infection and RT-qPCR assays. a Comparative analysis of JEV-infected C6/36 cells (right) versus mock-infected C6/36 cells (left). b JEV gene detection through RT-qPCR analysis

Additional file 3. Fig. S3 GETV Cell infection and RT-qPCR assays. a Comparative analysis of GETV-infected C6/36 cells (right) versus mock-infected C6/36 cells (left). b GETV gene detection through RT-qPCR analysis

Additional file 4. Fig. S4 Maximum likelihood phylogenetic tree based on RdRp sequences (≥ 200 amino acids) illustrating the detailed evolutionary relationships of viruses within the phylum *Duplornaviricota*

Additional file 5. Fig. S5 Maximum likelihood phylogenetic tree based on RdRp sequences (≥ 200 amino acids) illustrating the detailed evolutionary relationships of viruses within the phylum *Kitrinoviricota*

Additional file 6. Fig. S6 Maximum likelihood phylogenetic tree based on RdRp sequences (≥ 200 amino acids) illustrating the detailed evolutionary relationships of viruses within the phylum *Negarnaviricota*

Additional file 7. Fig. S7 Maximum likelihood phylogenetic tree based on RdRp sequences (≥ 200 amino acids) illustrating the detailed evolutionary relationships of viruses within the phylum *Pisuviricota*

Additional file 8. Table S1 Information on mosquito collection

Additional file 9. Table S2 Sequencing statistics of mosquito virome in this study

Additional file 10. Table S3 Classification and annotation of contig sequences from sequencing analysis performed with Kaiju, Kraken2, and MMseqs2

Additional file 11. Table S4 Results of BLAST searches (NR, NCBI Virus Genome, and ZVOER) for putative viruses after classification

Additional file 12. Table S5 Curated datasets of known and novel viruses identified in this study

Additional file 13. Table S6 Diversity of mosquito viromes across different countries or regions

## Data Availability

The raw sequencing data generated in this study have been deposited in the NCBI Sequence Read Archive (SRA) under BioProject accession PRJNA1212576. Complete genome sequences of ten Japanese encephalitis virus isolates are available in GenBank under accession numbers PV026202-PV026211. Representative sequences of Getah virus (GETV-GIII strain) and Banna virus (BAV-A2 strain) have been assigned GenBank accession numbers PV468710 and PV468711, respectively. Moreover, twenty-two newly assembled viral genome sequences using MEGAHIT software are publicly accessible through GenBank under accession numbers PV468710-PV468733.

## Abbreviations

COI: Cytochrome C oxidase subunit I
TPM: Transcripts per million
OTUs: Operational taxonomic units
RdRp: RNA-dependent RNA polymerase
RT-qPCR: Reverse transcription-quantitative PCR
ISVs: Insect-specific viruses
MBVs: Mosquito-borne viruses
PBS: Phosphate-buffered saline
FBS: Fetal bovine serum
ORF: Open reading frame
JEV: Japanese encephalitis virus
GETV: Getah virus
BAV: Banna virus
DENV: Dengue virus
GMNV: Guizhou mosquito novel virus
WNV: West Nile virus
ZIKV: Zika virus
