# Supplementary figures and images for: Unveiling viral diversity and dynamics in mosquitoes through metagenomic analysis in Guizhou Province, China
Source: Infect Dis Poverty. 2025 Jun 19;14:51. doi: 10.1186/s40249-025-01321-9 (PMC12178013; doi:10.1186/s40249-025-01321-9)

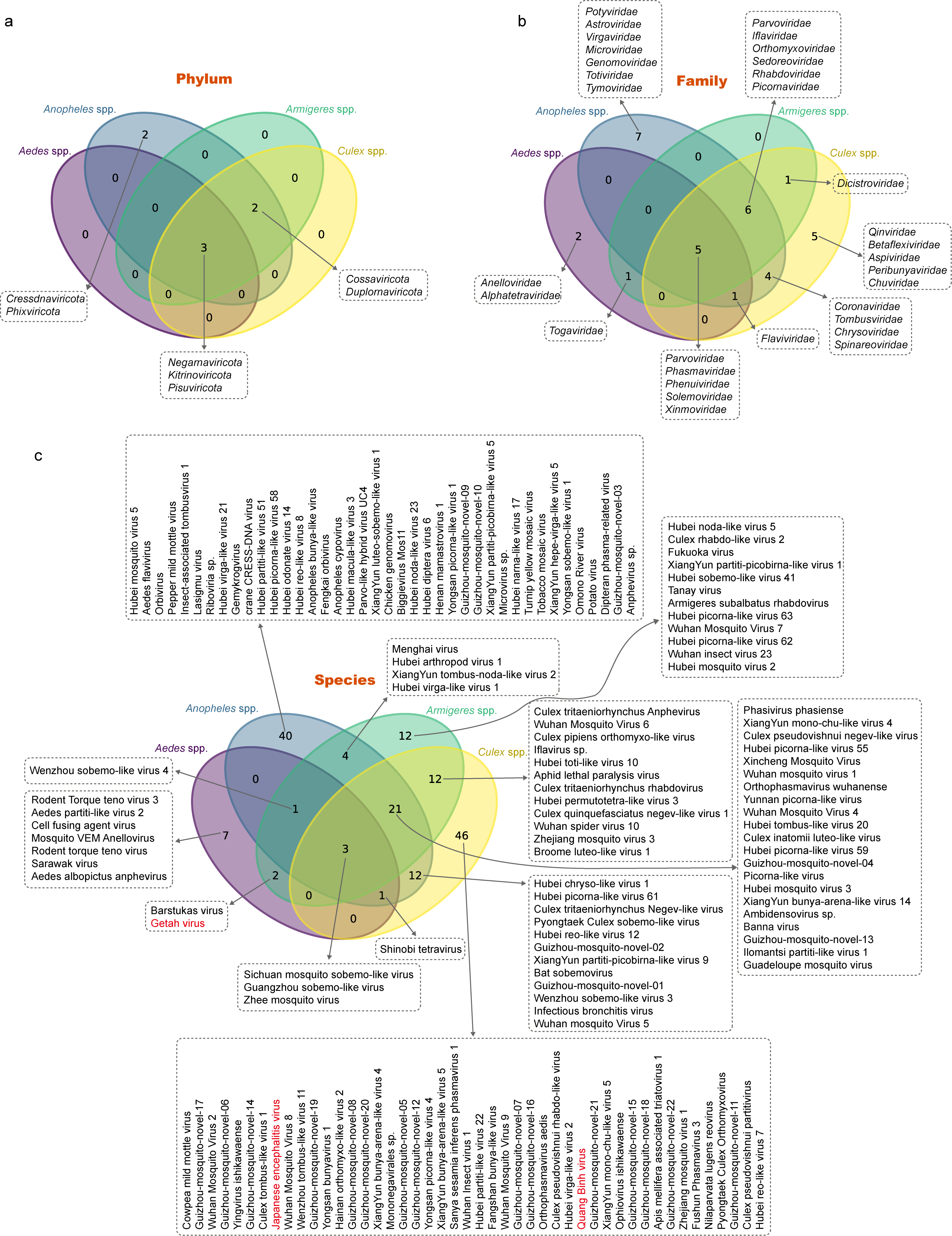

Supplement: Supplementary file 1 — Additional file 1. Fig. S1 Venn diagrams showing the overlap of viruses identified at the phylum a, family b, and species c levels. Numbers indicate the counts of intersecting or unique phyla, families, and species. In panel c, red-labeled viruses (Getah virus, Japanese encephalitis virus, and Quang Binh virus) represent species validated through cell isolation and gene sequencing [file 40249_2025_1321_MOESM1_ESM.tif]

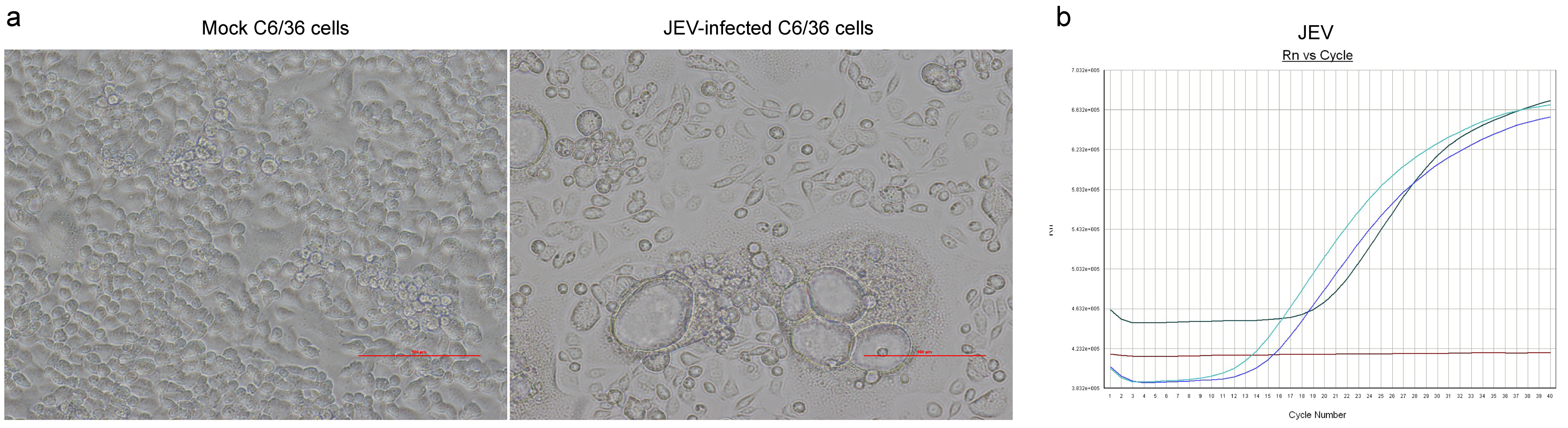

Supplement: Supplementary file 2 — Additional file 2. Fig. S2 JEV Cell infection and RT-qPCR assays. a Comparative analysis of JEV-infected C6/36 cells (right) versus mock-infected C6/36 cells (left). b JEV gene detection through RT-qPCR analysis [file 40249_2025_1321_MOESM2_ESM.tif]

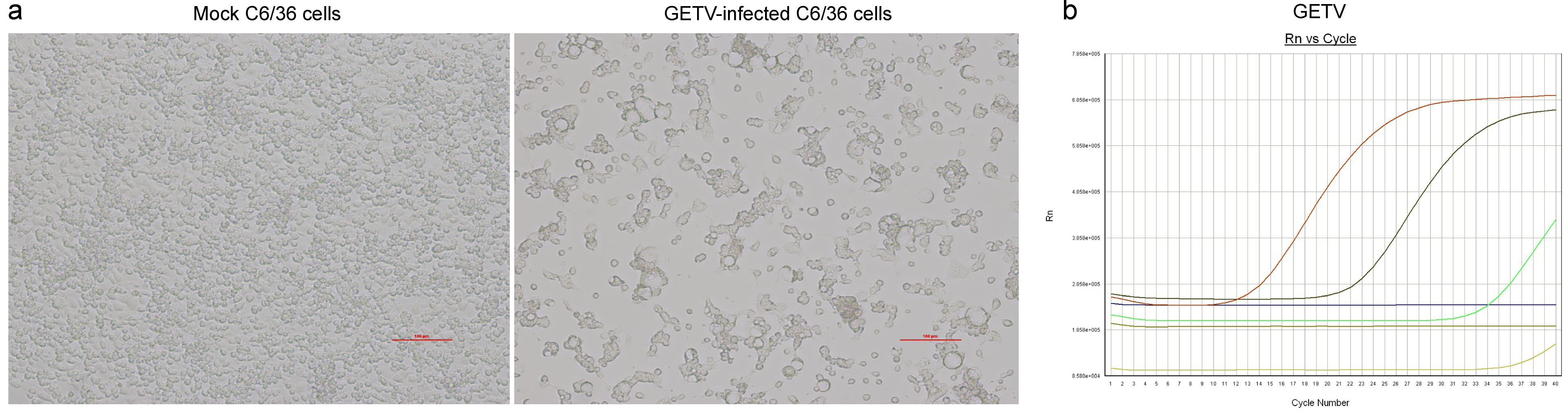

Supplement: Supplementary file 3 — Additional file 3. Fig. S3 GETV Cell infection and RT-qPCR assays. a Comparative analysis of GETV-infected C6/36 cells (right) versus mock-infected C6/36 cells (left). b GETV gene detection through RT-qPCR analysis [file 40249_2025_1321_MOESM3_ESM.tif]

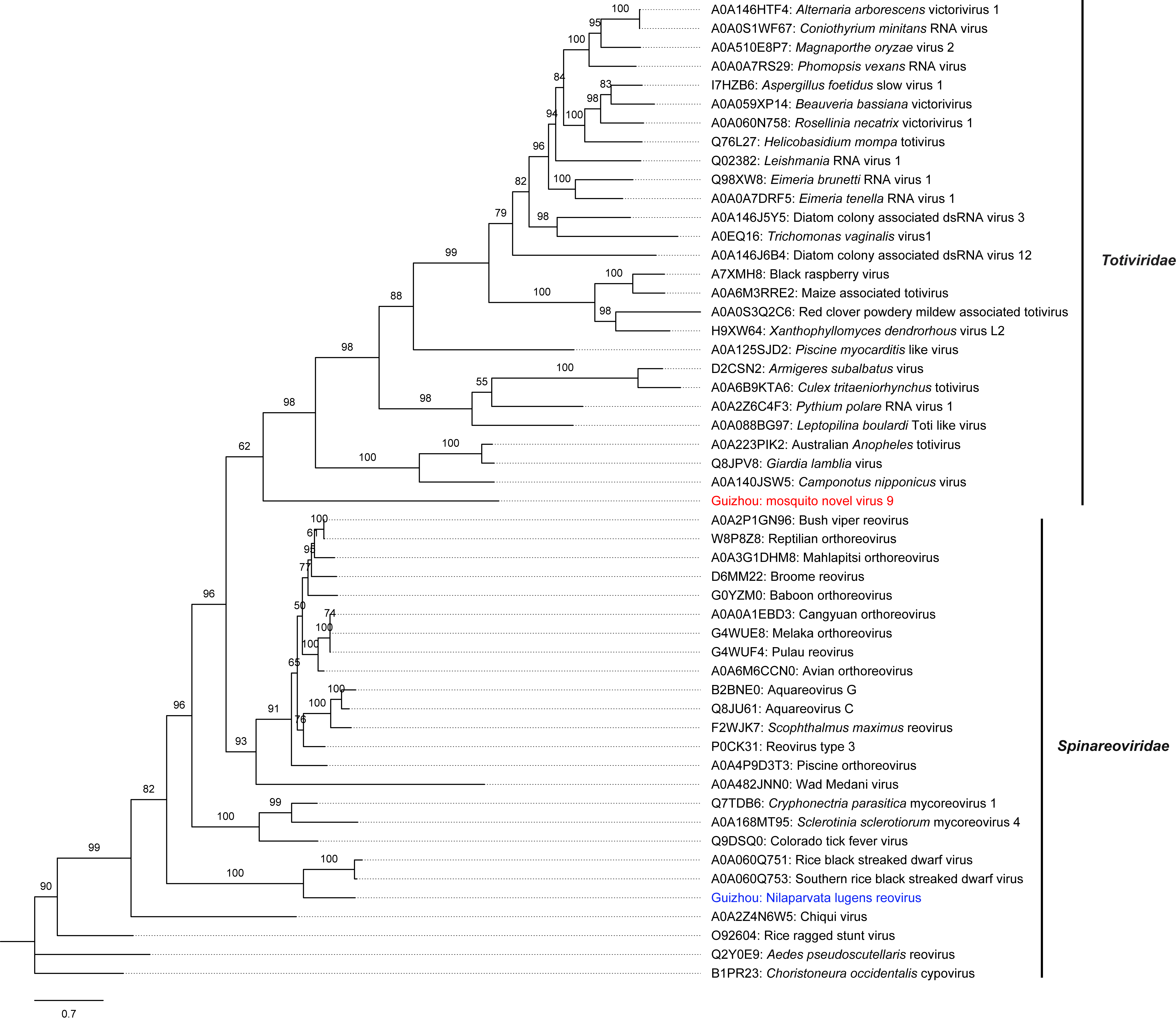

Supplement: Supplementary file 4 — Additional file 4. Fig. S4 Maximum likelihood phylogenetic tree based on RdRp sequences (≥ 200 amino acids) illustrating the detailed evolutionary relationships of viruses within the phylum Duplornaviricota [file 40249_2025_1321_MOESM4_ESM.tif]

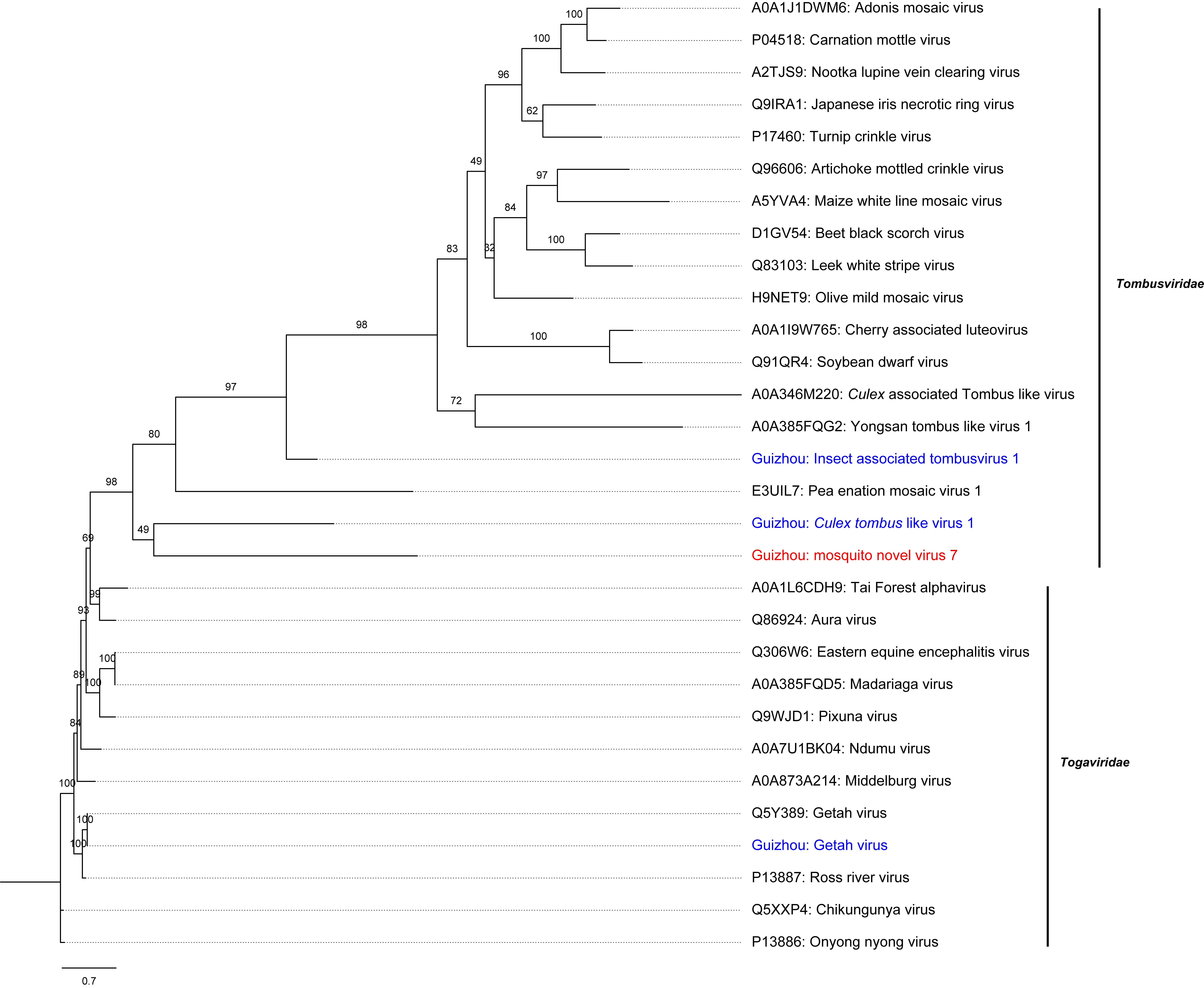

Supplement: Supplementary file 5 — Additional file 5. Fig. S5 Maximum likelihood phylogenetic tree based on RdRp sequences (≥ 200 amino acids) illustrating the detailed evolutionary relationships of viruses within the phylum Kitrinoviricota [file 40249_2025_1321_MOESM5_ESM.tif]

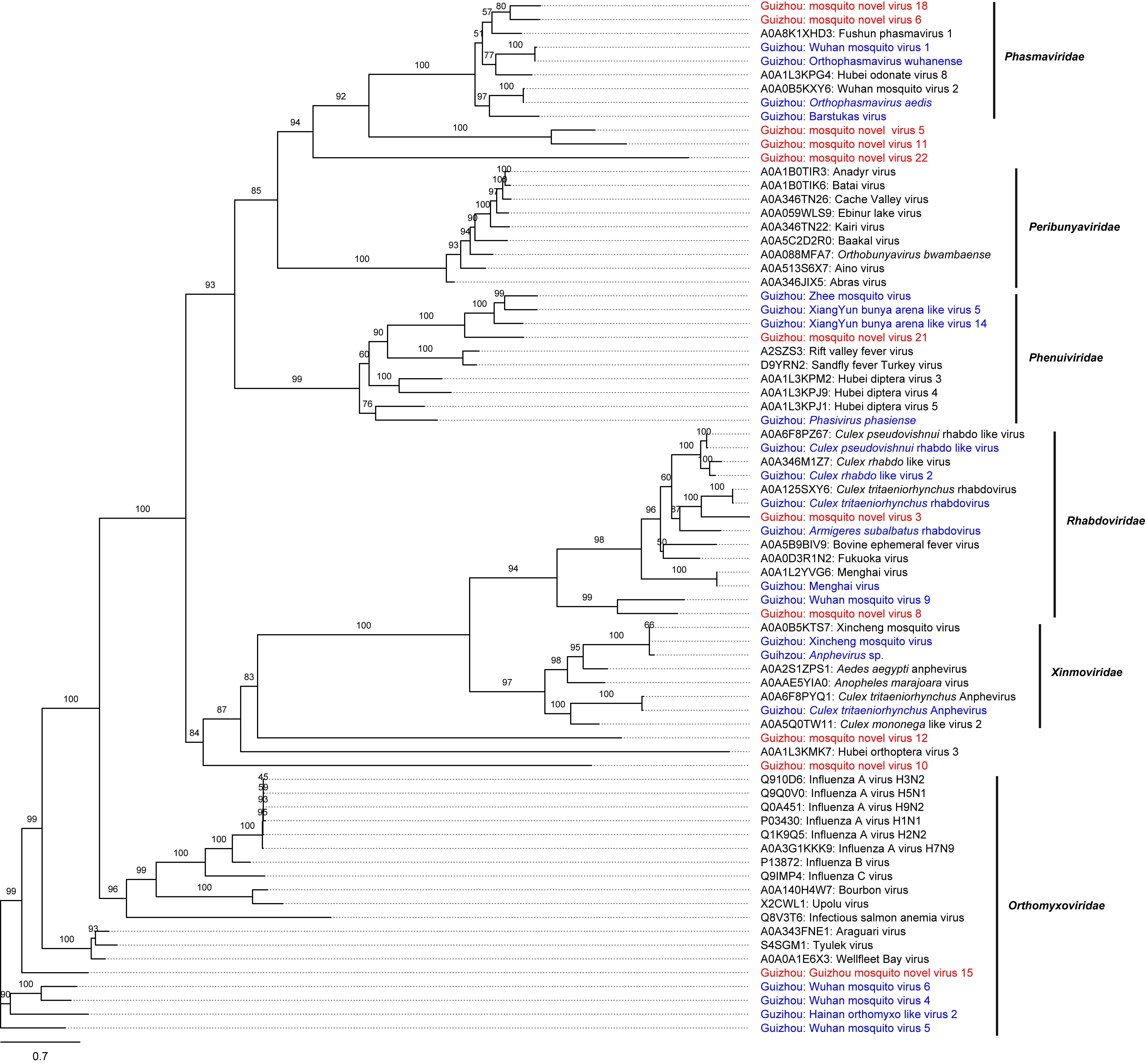

Supplement: Supplementary file 6 — Additional file 6. Fig. S6 Maximum likelihood phylogenetic tree based on RdRp sequences (≥ 200 amino acids) illustrating the detailed evolutionary relationships of viruses within the phylum Negarnaviricota [file 40249_2025_1321_MOESM6_ESM.tif]

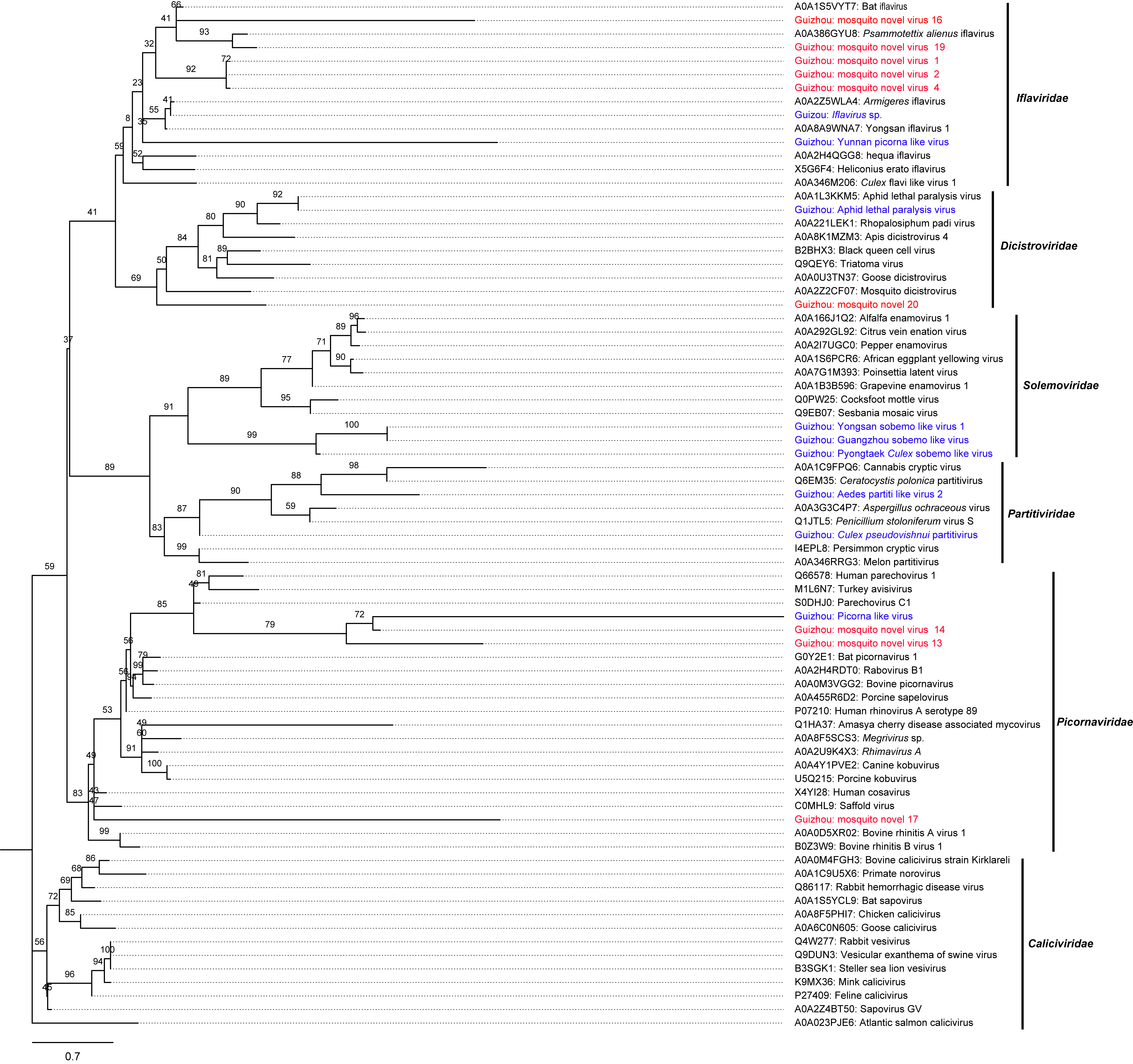

Supplement: Supplementary file 7 — Additional file 7. Fig. S7 Maximum likelihood phylogenetic tree based on RdRp sequences (≥ 200 amino acids) illustrating the detailed evolutionary relationships of viruses within the phylum Pisuviricota [file 40249_2025_1321_MOESM7_ESM.tif]
